# Computer-Aided Rational Engineering of Signal Sensitivity of Quorum Sensing Protein LuxR in a Whole-Cell Biosensor

**DOI:** 10.3389/fmolb.2021.729350

**Published:** 2021-08-13

**Authors:** Jinyu Li, Ruicun Liu, Yulu Chen, Shuxia Liu, Cheng Chen, Tuoyu Liu, Shan Yang, Yingtan Zhuang, Ruifu Yang, Yujun Cui, Yajun Song, Tao Wang, Yue Teng

**Affiliations:** ^1^ State Key Laboratory of Pathogen and Biosecurity, Beijing Institute of Microbiology and Epidemiology, Academy of Military Medical Sciences, Beijing, China; ^2^ School of Life Sciences, Tianjin University, Tianjin, China; ^3^ School of Basic Medical Sciences, Shandong University, Jinan, China

**Keywords:** LuxR, rational protein engineering, signal sensitivity, quorum sensing, gene circuit, biosensor

## Abstract

LuxR, a bacterial quorum sensing-related transcription factor that responds to the signaling molecule 3-oxo-hexanoyl-homoserine lactone (3OC6-HSL). In this study, we employed molecular dynamics simulation and the Molecular Mechanics Generalized Born Surface Area (MM-GB/SA) method to rationally identify residues in *Vibrio fischeri* LuxR that are important for its interaction with 3OC6-HSL. Isoleucine-46 was selected for engineering as the key residue for interaction with 3OC6-HSL-LuxR-I46F would have the strongest binding energy to 3OC6-HSL and LuxR-I46R the weakest binding energy. Stable wild-type (WT) LuxR, I46F and I46R variants were produced in *Escherichia coli* (*E. coli*) in the absence of 3OC6-HSL by fusion with maltose-binding protein (MBP). Dissociation constants for 3OC6-HSL from MBP-fusions of WT-, I46F- and I46R-LuxR determined by surface plasmon resonance confirmed the binding affinity. We designed and constructed a novel whole-cell biosensor on the basis of LuxR-I46F in *E. coli* host cells with a reporting module that expressed green fluorescent protein. The biosensor had high sensitivity in response to the signaling molecule 3OC6-HSL produced by the target bacterial pathogen *Yersinia pestis*. Our work demonstrates a practical, generalizable framework for the rational design and adjustment of LuxR-family proteins for use in bioengineering and bioelectronics applications.

## Introduction

Quorum sensing (QS) is a cell-to-cell communication mechanism involving extracellular signaling molecules that bacteria use to orchestrate collective behaviors (Bassler and Losick, 2006; Atkinson and Williams, 2009). In Gram-negative bacteria, acyl-homoserine lactones (acyl-HSLs) are the signaling molecules that mediate QS (Churchill and Chen, 2011). Acyl-HSLs are synthesized by members of the LuxI protein family, transported across the bacterial cell envelope, and recognized by LuxR-family sensor proteins (Egland and Greenberg, 1999; Fuqua et al., 2001). *Vibrio fischeri* LuxR acts as a transcriptional activator on binding 3-oxo-hexanoyl-homoserine lactone (3OC6-HSL) (Stevens et al., 1999; Chatterjee et al., 2005). The LuxI/LuxR QS system has been used by biological engineers to construct bacterial sensors (Steindler and Venturi, 2007), artificial bacterial consortia (Danino et al., 2010), and population synchronizers (Collins et al., 2006). High performance is required for such devices, including reliability, sensitivity, and adjustability. It is of great significance to explore methods to engineer systems to meet these requirements.

Directed evolution combined with random mutagenesis has successfully identified residues that strongly influence the signal specificity of LuxR (Chai and Winans, 2004; Hawkins et al., 2007; Rajput and Kumar, 2017). However, because of its intrinsically random output and time-consuming nature, directed evolution is limited in its ability to explore the relationships between protein sequence and function. This hinders the exploitation of LuxR in gene circuits. Structure-based molecular dynamics (MD) simulation (Ghanakota and Carlson, 2016; Rodriguez-Bussey et al., 2016) has also been used to identify residues that are critical for the affinity and sensitivity of LuxR for 3OC6-HSL (Collins et al., 2005; Tashiro et al., 2016; Kviatkovski et al., 2018), but in such cases, real proteins may not be readily available for biochemical validation or use. Here, we describe a multifaceted approach involving computational and biochemical analyses for the rational design of LuxR receptor proteins with tuned sensitivity for 3OC6-HSL and demonstrate their application in a whole-cell biosensor using a genetic circuit. This strategy is expected to promote the engineering of novel functional LuxR mutants.

## Materials and Methods

Common biochemical procedures (bacterial growth, site-directed mutagenesis, protein expression and purification, and determination of dissociation constants by surface plasmon resonance) are described in Supplementary Material.

### Molecular Structure Preparation and Molecular Dynamics

We selected the structure of the classical LuxR-family protein *Agrobacterium tumefaciens* TraR (PDB ID: 1L3L) as the template for MD simulations to obtain a stable structure of *V. fischeri* (also called *Aliivibrio fischeri*) LuxR. During the modeling and kinetic process, the LuxR protein was surrounded by TIP3P-type water molecules, and sodium and chloride ions were added to the system at an ion concentration of 150 mM to ensure that the molecules were in a normal physiological environment and the system maintained electrical neutrality. Visual Merchandising Design (VMD) software with MODELLER was used for modeling and visualization, and dynamic optimization was performed using the open-source kinetic calculation software NAnoscale Molecular Dynamic (NAMD) (Humphrey et al., 1996; Phillips et al., 2005). The Chemistry at Harvard Macromolecular Mechanics (CHARMM) force field was used for the kinetic process, the temperature of the system was 310 K, and the pressure was 1 bar (MacKerell et al., 1998 ; Teng et al., 2017). The kinetic process used a truncation function calculated from 13 to 15 Å. The MD simulation time was 100 ns.

### Molecular Docking

AutoDock software version 4.2 (http://autodock.scripps.edu) was used for molecular docking as previously described (Morris et al., 2009; Teng et al., 2019). For docking to apo- (i.e., ligand-free) LuxR, polar hydrogen atoms were added to LuxR and its nonpolar hydrogen atoms were merged using AutoDock Tools (http://mgltools.scripps.edu). The structure of apo-LuxR served as the receptor for docking with 3OC6-HSL. Binding energy was calculated as the sum of the intermolecular energy and the torsional free energy. AutoDockTools 1.5.6 software was used to prepare the semi-flexible docking procedures. The default 0.375 Å spacing was adopted for the grid box, and the volume was set as 32 × 32 × 32 Å. The key residues for the binding of LuxR and 3OC6-HSL were selected based on an extensive alignment of LuxR-family proteins from both a sequence and a structural perspective, then selected as the docking pocket. The number of runs was set to 100.

### MM-GB/SA Calculations

Binding free energy between LuxR and 3OC6-HSL was calculated using the Molecular Mechanics Generalized Born Surface Area (MM-GB/SA) method (Genheden and Ryde, 2015; Teng et al., 2019). For each system, 1,000 snapshots without water and ions were extracted from the last 20 ns of the equilibration trajectory.

Binding free energy (*∆G*_*bind*_) was calculated as:

$$\Delta G_{bind}=G_{complex}-\left(G_{receptor}+G_{ligand}\right)$$ (1)

where *G*_*complex*_, *G*_*receptor*_, and *G*_*ligand*_ are the binding free energies of the complex, the receptor, and the ligand, respectively.

Free energy (*∆G*_*TOT*_) was calculated using the equations:

$$\Delta G_{TOT}=EMM+G_{sol}-TDS$$ (2)

$$\Delta EMM=\Delta E_{ele}+\Delta E_{vd}W$$ (3)

$$\Delta G_{sol}=\Delta G_{polar}+\Delta G_{nonpolar}$$ (4)

$$G_{nonpolar}=g\times SASA+b$$ (5)

where *∆EMM*, *∆G*_*sol*_, and *TDS* refer to molecular mechanics free energy, solvation free energy, and entropy in the gas phase, respectively. *∆EMM* can be divided into electrostatic interactions (*∆E*_*ele*_) and van der Waals interactions (*∆E*_*vd*_ *W*). *∆G*_*sol*_ can be divided into polar contributions (*∆G*_*polar*_) and nonpolar contributions (*∆G*_*nonpolar*_). *∆G*_*polar*_ is calculated using the Generalized Born method. *∆G*_*nonpolar*_ is considered to be proportional to the molecular solvent-accessible surface area (SASA) buried upon binding, in which *g* and *b* were set to the default values of 0.0072 kcal (mol·Å^2^) and 0.00, respectively.

### Overexpression and Purification of Wild-Type and Mutant LuxR–MBP Fusion Proteins

The pMAT9S-based plasmids for expressing LuxR-WT–MBP fusions and the corresponding mutants were respectively transformed into *E. coli* BL21 (DE3) competent cells. Cultures were grown in LB medium containing ampicillin at 310 K until the optical density at 600 nm reached 0.8. Isopropyl-β-D-thiogalactopyranoside was then added to a final concentration of 0.4 mM and culture was continued at 289 K for 16 h. Thereafter, cells were harvested by centrifugation and the bacterial pellets were resuspended in phosphate-buffered saline (140 mM NaCl, 10 mM Na2HPO4, 2.7 mM KCl, 1.8 mM KH2PO4, pH 7.3) supplemented with 15% glycerol. After high-pressure crushing at 277 K, bacterial lysates were centrifuged at 18,000 × g for 40 min at 277 K and the pellet was discarded. The supernatant was loaded onto a disposable column containing amylose resin (New England Biolabs, cat. no. E8021S) to purify MBP-tagged WT or mutant LuxR. The fusion proteins were eluted using 20 mM maltose dissolved in resuspension buffer and further purified by anion-exchange chromatography using a HiTrap Q column (GE Healthcare) with a linear gradient from 25 to 250 mM NaCl in 20 mM Tris-HCl (pH 8.0) and 15% glycerol, followed by gel filtration chromatography using a Superdex-200 column (GE Healthcare) in resuspension buffer.

### Surface Plasmon Resonance

SPR experiments were performed on a Biacore T200 SPR system (GE Healthcare) at 25°C in PBS-P (20 mM phosphate buffer with 2.7 mM KCl, 0.137 mM NaCl, and 0.05% surfactant P20). MBP-stabilized fusions of LuxR or its variants in PBS-P were respectively immobilized on CM5 chips by amine coupling. After twofold serial dilution of 3OC6-HSL in 5% (v/v) dimethylsulfoxide (DMSO), it was injected into the flow cell for 60 s at a flow-rate of 30 μl/min for association, followed by a 60-s dissociation phase. A high DMSO concentration was found to be necessary for solubility of 3OC6-HSL, helping to minimize nonspecific interactions and aggregation on the chip. The noise effect resulting from differences in DMSO concentration was corrected over a limited range (4.5–5.8%) of standards. Background binding to blank immobilized flow cells was subtracted, and equilibrium dissociation constant (*Kd*) values were calculated using the steady-state affinity model in BIAcore T200 Evaluation Software v.3.2.

### Gene Circuit Construction

Plasmid constructs were assembled to comply with the BioBrick RCF standard using a variety of molecular cloning techniques. Biological parts were acquired from the Registry of Standard Biological parts or produced by chemical synthesis. The constitutive promoter P_Con_ (BBa_J23104) with a ribosome-binding site (RBS; BBa_B0034) was inserted upstream of the synthetic *V. fischeri luxR* gene as the LuxR sender module. RBS (BBa_B0034)–GFP (BBa_E0040)–terminator (BBa_B0015) was inserted downstream of P_lux_ [a LuxR-HSL-regulated promoter (BBa_C0062)] as the LuxR receiver module. The modules were assembled into vector pSB1A3.

### Analysis of Engineered LuxR in a Whole-Cell Biosensor Using a Genetic Circuit

Overnight *Escherichia coli* cultures (see Supplementary Methods) were diluted 1000-fold into fresh LB medium containing 100 μg/ml ampicillin and transferred to 1.5-ml centrifuge tubes containing different concentrations of 3OC6-HSL (0, 10^−6^, 10^−7^, 10^−8^, 10^−9^, 10^−10^, 10^−11^, 10^−12^ M). Samples were incubated at 37°C for 8 h, and then transferred to a 96-well microplate and fluorescence was measured using a Synergy H1 hybrid Reader (excitation, 488 nm; emission, 518 nm). Cell densities were measured optically at 600 nm. Mean fluorescence was calculated from at least three biological replicates. In addition, the above 1:1,000 bacterial dilution and 3OC6-HSL (10^−6^, 10^−7^ M) were added to Corning Black Costar transparent bottom 96-well plates. The samples were incubated in the Synergy H1 hybrid Reader continuously for 8 h (807 rpm, orbital shaking), and the fluorescence intensity and OD_600_ were analyzed at 30-min intervals.

The GFP production of the whole-cell biosensor incubated with culture supernatants of bacterial pathogens was measured as noted below. Overnight *E. coli* cultures (see Supplementary Methods) were diluted 1:100 and further incubated to logarithmic growth phase in the same conditions. Biosensor cells were collected by centrifugation at 3,000 × g for 4 min and resuspended in an appropriate volume of LB medium to make the OD_600_ = 1. For the detection of native 3OC6-HSL, sterile culture supernatants of *Yersinia pestis* (*Y. pestis*), *Y. pestis* Δ*ypeRI* Δ*yspRI*, *E. coli* strain MG1655, and *Klebsiella pneumoniae* (*K. pneumoniae*) were collected at 8,000 × g for 5 min and co-cultured with biosensor cells (volume ratio 15: 1) for 11 h at 37°C. We also determined the sensitivity by incubating biosensor cells with sterile culture supernatants of *Y. pestis* taken from cultures with optical densities 0.2, 0.4, 0.6, 1.0, and 1.5. All GFP fluorescence intensity and OD_600_ measurements were made at 30-min intervals using a microplate reader.

## Results and Discussion

### MD Simulation and Binding Site Analysis of the LuxR-3OC6-HSL Complex

**FIGURE 1:**
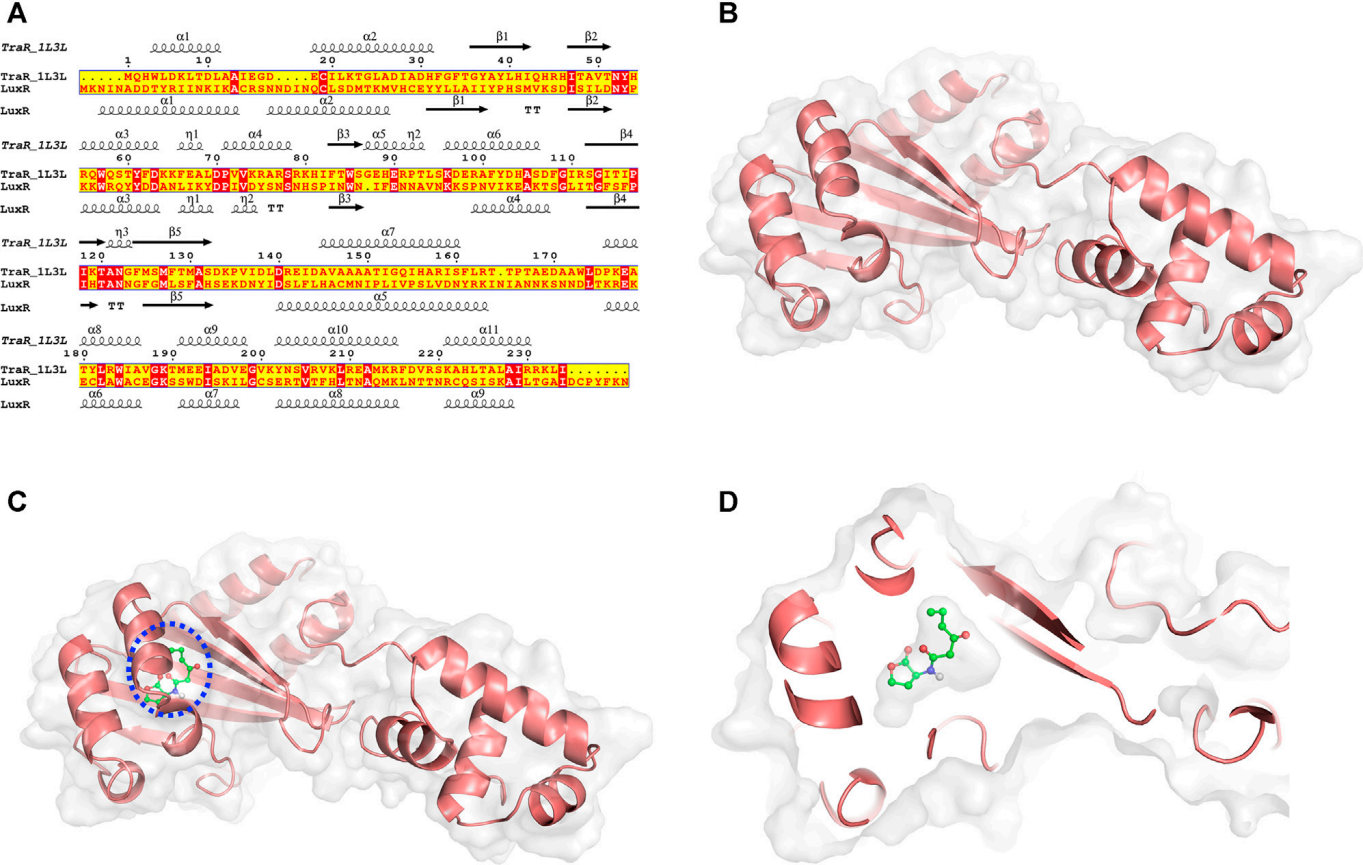
MD simulations and structural conformations of the *V. fischeri* LuxR. **(A)** Sequence alignment of Agrobacterium tumefaciens TraR (PDB ID: 1L3L) and Vibrio fischeri LuxR (DDBJ accession no. AF170104). Conserved amino acid residues are shown in red. **(B)** Overall modelled structure of *V. fischeri* LuxR. **(C)** Overall conformation and **(D)** close-up view of the LuxR–3OC6-HSL composite structure. The protein is displayed as a cartoon, and 3OC6-HSL is displayed using stick representation.

Structural modeling of *V. fischeri* LuxR (DDBJ accession no. of the coding sequence AF170104) was performed by MD simulation with the LuxR-family protein *A. tumefaciens* TraR (PDB ID: 1L3L) as the template (Figure 1). The crystal structure of TraR has been obtained by X-Ray diffraction at 1.66 Å resolution. The resulting LuxR structure is depicted in Figure 1B, and the quality of the structure was confirmed by Ramachandran plot analysis (Supplementary Figure S1). Using this structure of LuxR generated as the acceptor molecule and 3OC6-HSL from the TraR crystal structure as the ligand, the semi-flexible docking method in Autodock software was applied to perform molecular docking calculations (Figures 1C,D). PROCHECK software was used to validate the quality of the final LuxR*–*3OC6-HSL structure; a Ramachandran plot revealed that all residues of the LuxR*–*3OC6-HSL complex were in favored or allowed regions (91.5 and 8.5% respectively), indicating that the model of the LuxR*–*3OC6-HSL complex was of good quality (Supplementary Figure S2).

We selected a series of structural states, from 50, 75, and 100 ns (Supplementary Figure S3) in the MD process, to validate the stability of the final LuxR*–*3OC6-HSL complex structure. As shown in Supplementary Figure S3B, subtle fine-tuning of the position of 3OC6-HSL occurred as the molecular simulation progressed. A final and stable location for the 3OC6-HSL molecule was achieved, in which 3OC6-HSL exactly fitted the binding cavity of LuxR. Based on analysis of the interactions between LuxR and 3OC6-HSL, residues including L42, I58, Y62, W66, D70, and D79 were found to constitute the binding pocket of LuxR. Y62 and D79 form hydrogen bonds with 3OC6-HSL, while W66, I58 and the other residues help to stabilize 3OC6-HSL mainly via hydrophobic interactions.

### Identification and Verification of I46 as a Key Residue in the LuxR-3OC6–HSL Complex

**FIGURE 2:**
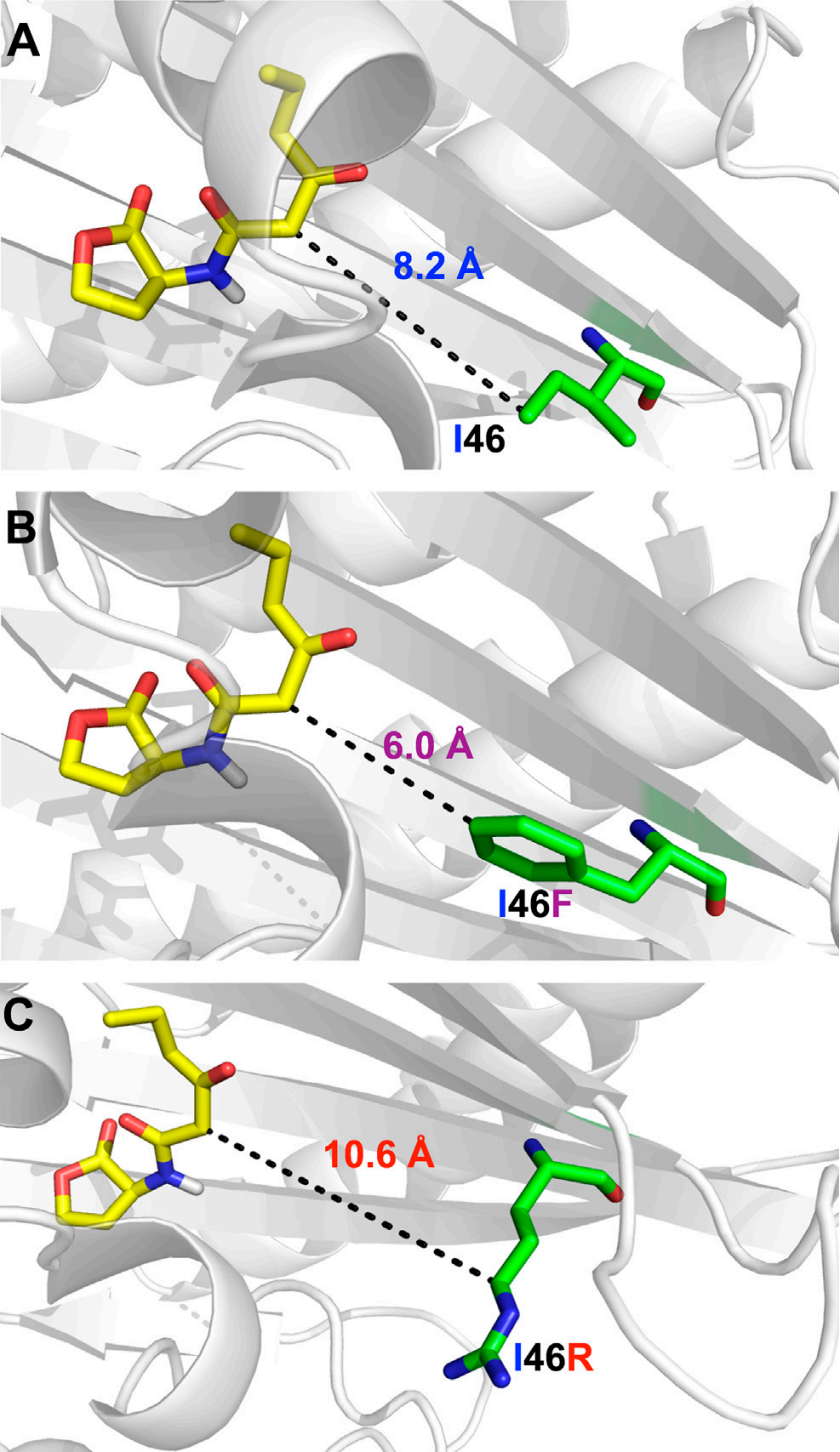
The structural relationship between LuxR residue I46 **(A)**, mutant I46F **(B)** and I46R **(C)** and bound 3OC6-HSL (residue backbones shown in green, 3OC6-HSL backbone in yellow).

Based on the above analysis of the interaction profile between LuxR and 3OC6-HSL, we employed the MM-GB/SA method to calculate energies for the key binding sites on LuxR of 3OC6-HSL (Supplementary Figure S4A). Interestingly, I46, a residue >8 Å from the binding site (Figure 2) that is not conserved in *A. tumefaciens* TraR (Figure 1), had a marked effect on the binding potential between LuxR and 3OC6-HSL. In the structural model, we mutated residues that affected 3OC6-HSL binding into the other naturally occurring proteinaceous amino acids and docked the 3OC6-HSL molecule into each mutant using semi-flexible docking, MD, and kinetic calculations. The change in docking energy relative to the wild-type (WT) protein served to indicate the importance of each site (Supplementary Figure S4B). When I46 was mutated, the heatmap indicated that LuxR-I46F would display the strongest binding energy to 3OC6-HSL, and I46R the weakest interaction (Supplementary Figure S4B), i.e., that mutation LuxR-I46F would improve the response of LuxR to 3OC6-HSL compared with the WT protein, and mutation LuxR-I46R would weaken it. LuxR was thus rationally engineered at position 46.

Because of the tendency of LuxR-family transcription factors to aggregate in the absence of HSLs (Qin et al., 2000), no direct biochemical characterization of the binding affinity between LuxR and 3OC6-HSL has been reported, except for a roughly calculated dissociation constant retrieved from a model fitted to luminescence data (Colton et al., 2015). Here, we applied the rigid MBP technique, which has enabled the crystallization and structural determination of numerous proteins, to production of stable apo-LuxR. In this approach, a rigid helix at the C-terminus of MBP constrains the flexibility of attached recombinant proteins and thereby improves their solubility (Waugh, 2016).

**FIGURE 3:**
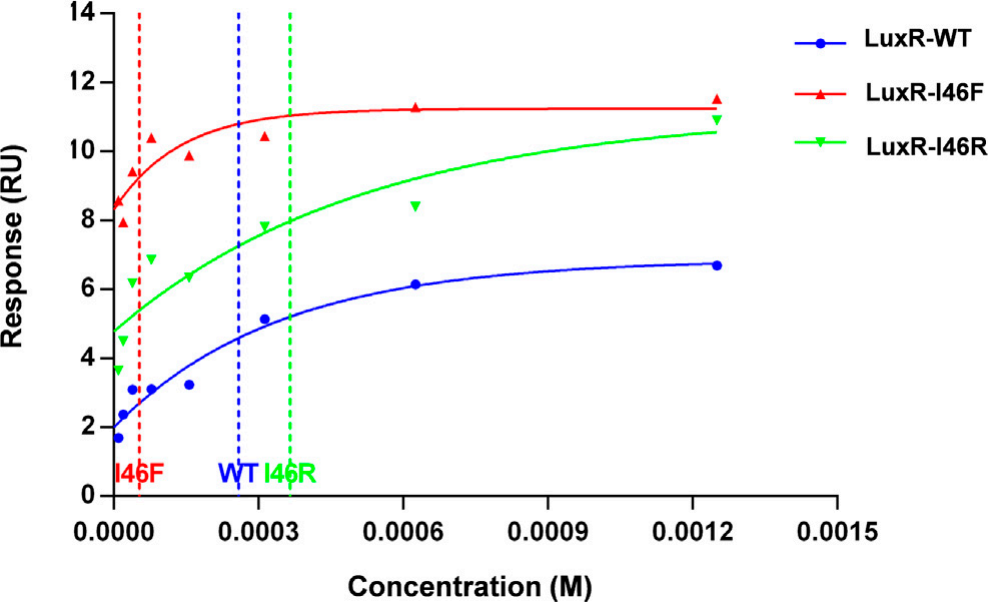
Surface plasmon resonance characterization of binding of 3OC6-HSL to maltose-binding protein fusions of residues 1–179 of WT *V. fischeri* LuxR, I46F and I46R mutants.

Applying this method, we produced a stable, soluble, apo-form of *V. fischeri* LuxR (residues 1–179, the N-terminal ligand-binding domain) as a fusion with MBP. We determined the dissociation constants for 3OC6-HSL binding to the MBP-fused LuxR and the LuxR-I46F and LuxR-I46R mutants by surface plasmon resonance (Figure 3; one-phase dissociation fitting). The dissociation constant values were 258.5 μM (the MBP-fused LuxR and 3OC6-HSL), 52.8 μM (the MBP-fused LuxR-I46F and 3OC6-HSL), and 365.0 μM (the MBP-fused LuxR-I46R and 3OC6-HSL), which indicated that—in accordance with the docking energy calculations—I46F mutation of LuxR increased the sensitivity of LuxR for 3OC6-HSL, and I46R decreased it.

### Application of LuxR-I46 Mutants in a Whole-Cell Biosensor

**FIGURE 4:**
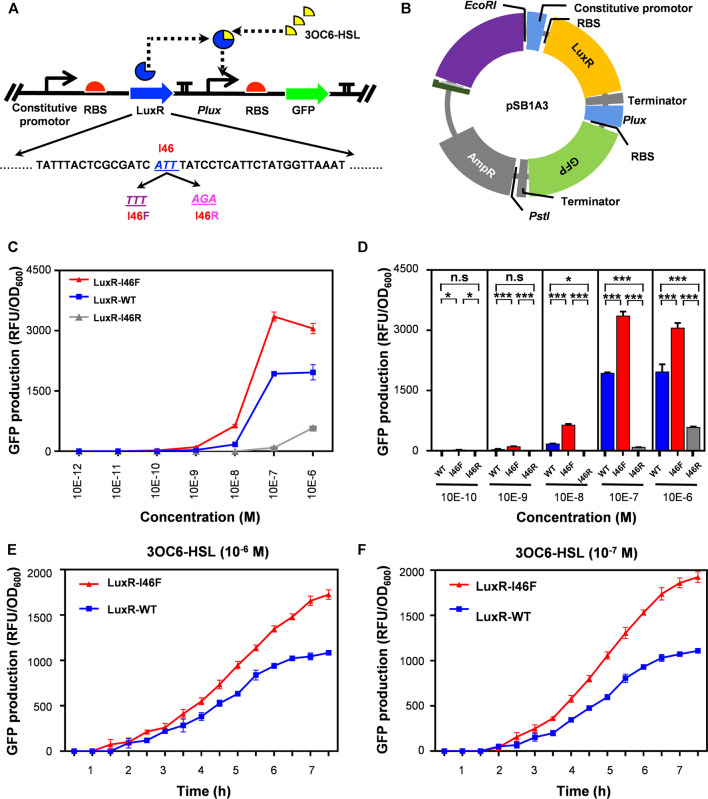
Gene circuits applying *V. fischeri* LuxR with modulated sensitivity to 3OC6-HSL. **(A)** Design of synthetic gene circuit using components from the Registry of Standard Biological parts, LuxR as the sensor, and GFP as the reporter. **(B)** Plasmid construction. **(C,D)** Plasmids encoding gene circuits including WT or mutant forms of LuxR were transformed into Escherichia coli DH5α, and fluorescence was measured to determine the response of LuxR to 3OC6-HSL. The data were first corrected by subtracting the negative control fluorescence, and then normalized by bacterial density (OD_600_). **(E,F)** Curves of relative fluorescence intensity change with temperature at  ~ 1 × 10^–6^ M **(E)** and  ˜ 1 × 10^–7^ M **(F)** 3OC6-HSL. All the results are the means of triplicate experiments.

We constructed a novel whole-cell biosensor using the various forms of LuxR (WT, I46F and I46R mutants) with different sensitivities to 3OC6-HSL in a one-plasmid system (Figures 4A,B). One module, P_Con_–luxR, expressed the transcription factor LuxR under the control of a constitutive promoter as an intermediate for signal transduction. The following reporting module, P_lux_–GFP, expressed GFP under the control of the P_lux_ promoter. The amount of GFP production from P_lux_ is dependent on the degree of transcriptional activation that occurs as a result of functional LuxR binding to a specific acyl-HSL signaling molecule.

Bioassay using these gene circuit constructs allowed us to quantitatively characterize the different forms of LuxR by GFP production rates in response to varying concentrations of 3OC6-HSL. LuxR-I46F displayed a sensitive and stable response to 3OC6-HSL: this system emitted significant fluorescence at 3OC6-HSL concentrations ∼1 × 10^–10^ M, while the construct with LuxR-WT did not respond until the concentration was 1 × 10^−9^ M, and that with LuxR-I46R did not respond until it was 1 × 10^–7^ M (Figures 4C,D). In terms of the response time, the gene circuit based on LuxR-I46F also displayed sensitivity and stability: The LuxR-I46F system showed a significant fluorescence difference for the first time at 1.5 h with ∼1 × 10^−6^ M 3OC6-HSL, and at 2.5 h with ∼1 × 10^−7^ M 3OC6-HSL (Figures 4E,F).

To determine the ability of whole-cell biosensors based on LuxR-WT and LuxR-I46F to detect 3OC6-HSL produced by bacterial pathogens, the biosensor cells were incubated in the culture supernatant of *Yersinia pestis. Y. pestis* is the causative agent of plague, a highly virulent bacterium, and a serious threat to public health. The type I quorum sensing system of *Y. pestis* includes two sets of LuxI/LuxR QS proteins, namely YpeI/YpeR and YspI/YspR. Signal molecules including 3OC6-HSL are synthesized by YpeI/YspI. The whole-cell biosensors based on LuxR-WT and LuxR-I46F were incubated with culture supernatants of different bacterial strains in logarithmic growth phase (*Y. pestis*, *Y. pestis* Δ*ypeRI* Δ*yspRI, K. pneumoniae* and *E. coli* MG1655). Only the culture supernatant of *Y. pestis* contained the target signal molecules; the latter three bacterial culture supernatants served as negative controls. Knockout of YpeI/YpeR and YspI/YspR does not affect the growth of *Y. pestis* (see Supplementary Figure S6 for growth curves of the various bacteria).

**FIGURE 5:**
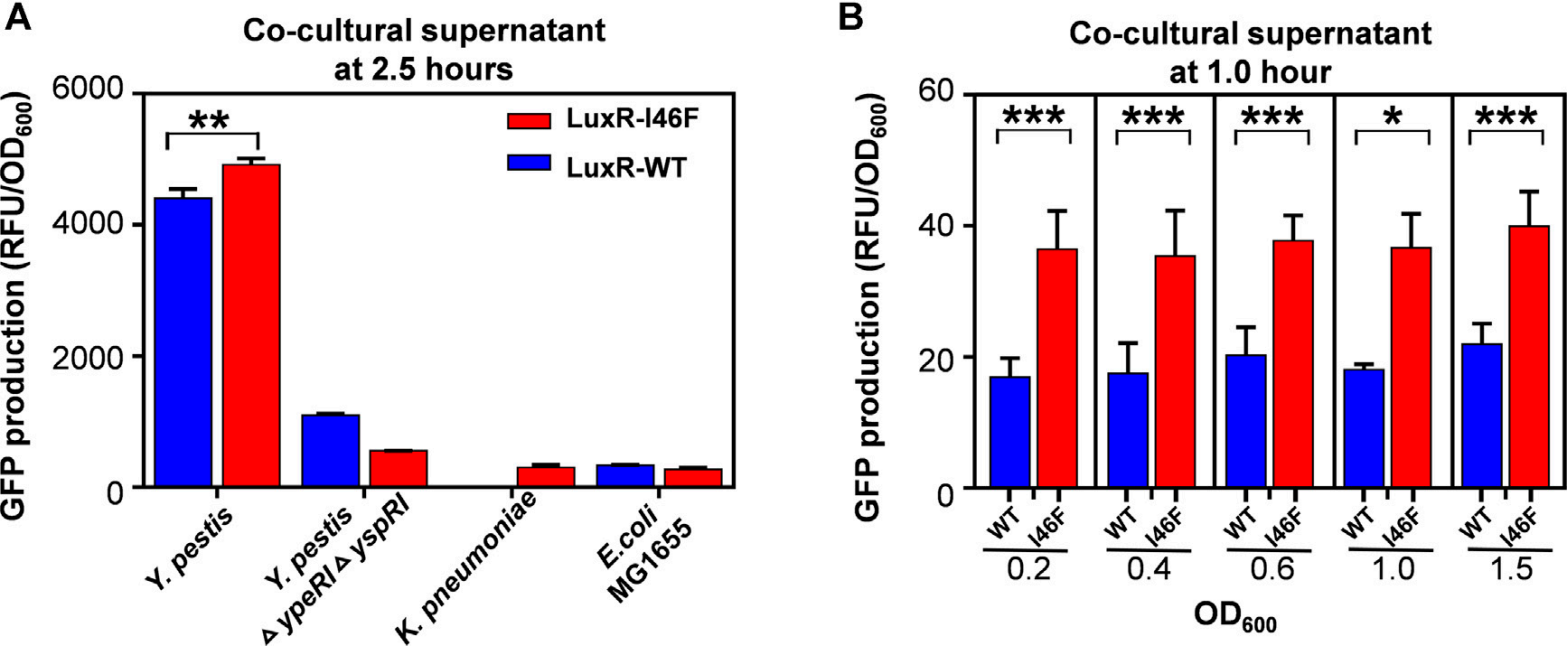
**(A)** Relative fluorescence intensity of the biosensors based on LuxR-WT and LuxR-I46F incubated in culture supernatant of *Yersinia pestis* (OD_600_ = 1.4), *Y. pestis* Δ*ypeRI* Δ*yspRI* (OD_600_ = 1.4)*, K. pneumoniae* (OD_600_ = 0.7), or *E. coli* strain MG1655 (OD_600_ = 0.7) for 2.5 h. **(B)** Relative fluorescence intensity of the biosensors based on LuxR-WT and LuxR-I46F incubated in culture supernatant of *Y. pestis* or *Y. pestis* Δ*ypeRI* Δ*yspRI* (OD_600_ = 0.2, 0.4, 0.6, 1.0, or 1.5) for 1 h. The results are the means of triplicate experiments.

The whole-cell biosensors based on LuxR-I46F displayed a sensitive and stable response to the culture supernatant of *Y. pestis*: Compared with the negative controls, both LuxR-WT and LuxR-I46F biosensors can detect 3OC6-HSL in the supernatant of *Y. pestis* containing LuxI/LuxR QS systems. After incubating for 2.5 h, the relative fluorescence intensity of the LuxR-I46F biosensor was significantly higher than that of the LuxR-WT biosensor (Figure 5). The difference in the relative fluorescence intensity of the LuxR-I46F and LuxR-WT biosensors increased significantly with incubation time (Supplementary Figure S5). In addition, the biosensor based on LuxR-I46F can respond accurately and quickly to the culture supernatant of *Y. pestis* in the initial growth stage [OD_600_ = 0.2 (representing incubation for 0.5 h)] (Figure 5B). In all, the results provide solid evidence that the whole-cell biosensor based on LuxR-I46F can sensitively and rapidly detect native 3OC6-HSL produced by a bacterial pathogen.

## Conclusion

Previous methods for development of LuxR mutants have significant disadvantages and cannot fully exploit the biotechnological potential of this sensor. In this work, we demonstrate a robust multi-method strategy for rational design and production of LuxR mutants for use in gene circuits, sensors, and QS-related applications. Notably, the method was applied to a LuxR for which no high-resolution structure is available. Via computational structural modelling, free energy-based analysis, stable MBP-fusion protein expression, and biochemical verification, mutants of LuxR were developed for use in functionally effective gene circuits. The key residue identified, I46, was not conserved in the template used to build the structural model, so would likely not be picked up by conventional sequence or structural analyses. In the gene circuit, the LuxR mutants tuned the response of the fluorescent reporter to the signaling molecule 3OC6-HSL: the construct was sensitive to 1 × 10^–10^ M 3OC6-HSL if the LuxR-I46F variant was used, and 1 × 10^–7^ M if LuxR-I46R was incorporated.

In addition, we designed a whole-cell biosensor based on LuxR-I46F and studied the feasibility of detecting small molecules produced by pathogenic bacteria. Compared with chemical or physical analytical techniques, our whole-cell biosensor has the advantages of low cost, strong portability, environmental compatibility, and rapid detection (Lindler et al., 2001; Meyer et al., 2007; Matero et al., 2009). The whole-cell biosensor can generate significant signals in the early growth of *Y. pestis* (i.e., after 1 h of incubation). The biosensor could provide a simple and reliable alternative for timely and immediate detection of target signal molecules of bacterial pathogens.

There are limitations of this work, notably that only a single QS protein and a single ligand were analyzed. It is likely that a greater sensitivity range can be generated by screening QS sensors and their ligands from a range of bacteria, which would also access a wider range of activator molecules. Moreover, only a single amino acid of LuxR was mutated in this study. By iteratively screening and mutating multiple residues, the sensitivity difference between high- and low-affinity mutants, and the maximum sensitivity, may be substantially amplified. In addition, it was only confirmed that the biosensors constructed in this work can detect *Y. pestis*. In future studies, the scope of detection of target pathogens needs to be expanded. The strategy demonstrated in this report can be generalized to the rational design of LuxR-family proteins for use as novel components in bioengineering applications.

## Data Availability

The original contributions presented in the study are included in the article/Supplementary Material, further inquiries can be directed to the corresponding authors.
