# Supplementary material for: Computer-Aided Rational Engineering of Signal Sensitivity of Quorum Sensing Protein LuxR in a Whole-Cell Biosensor
Source: Front Mol Biosci. 2021 Aug 13;8:729350. doi: 10.3389/fmolb.2021.729350 (PMC8415086; doi:10.3389/fmolb.2021.729350)
Supplement: Supplementary file 1 [file DataSheet1.docx]

**Supplementary Materials**

**Computer-aided rational engineering of signal sensitivity of quorum sensing protein LuxR in a whole-cell biosensor**

Jinyu Li^a,b,¶^, Ruicun Liu^a,¶^, Yulu Chen^a,¶^, Shuxia Liu^a^, Cheng Chen^b^, Tuoyu Liu^a^, Shan Yang^a^, Yingtan Zhuang^c^, Ruifu Yang^a^, Yujun Cui^a^, Yajun Song^a,†^, Tao Wang^b,†^, Yue Teng^a,†^

^a^ State Key Laboratory of Pathogen and Biosecurity, Beijing Institute of Microbiology and Epidemiology, Beijing 100071, China;

^b^ School of Life Sciences, Tianjin University, Tianjin 300110, China;

^c^ School of Basic Medical Sciences, Shandong University, Jinan, Shandong 250012, China.

^¶^ These authors contributed equally to this work.

† Correspondence should be addressed to:

Yue Teng, State Key Laboratory of Pathogen and Biosecurity, Beijing Institute of Microbiology and Epidemiology, 20 Dong-Da Street, Fengtai District, Beijing 100071, China. Telephone: +86-10-68164807; Fax: +86-10-68167357; Email: [yueteng@sklpb.org](mailto:yueteng@sklpb.org)

Tao Wang, School of Life Sciences, Tianjin University, 92 Weijin Road, Nankai District, Tianjin 300110, China. Telephone: +86-18622676379; Email: wangtaobio@tju.edu.cn

Yajun Song, State Key Laboratory of Pathogen and Biosecurity, Beijing Institute of Microbiology and Epidemiology, 20 Dong-Da Street, Fengtai District, Beijing 100071, China. Telephone: +86-10-68164807; Fax: +86-10-68167357; Email: songyajun88@aliyun.com

**Supplementary Figures**


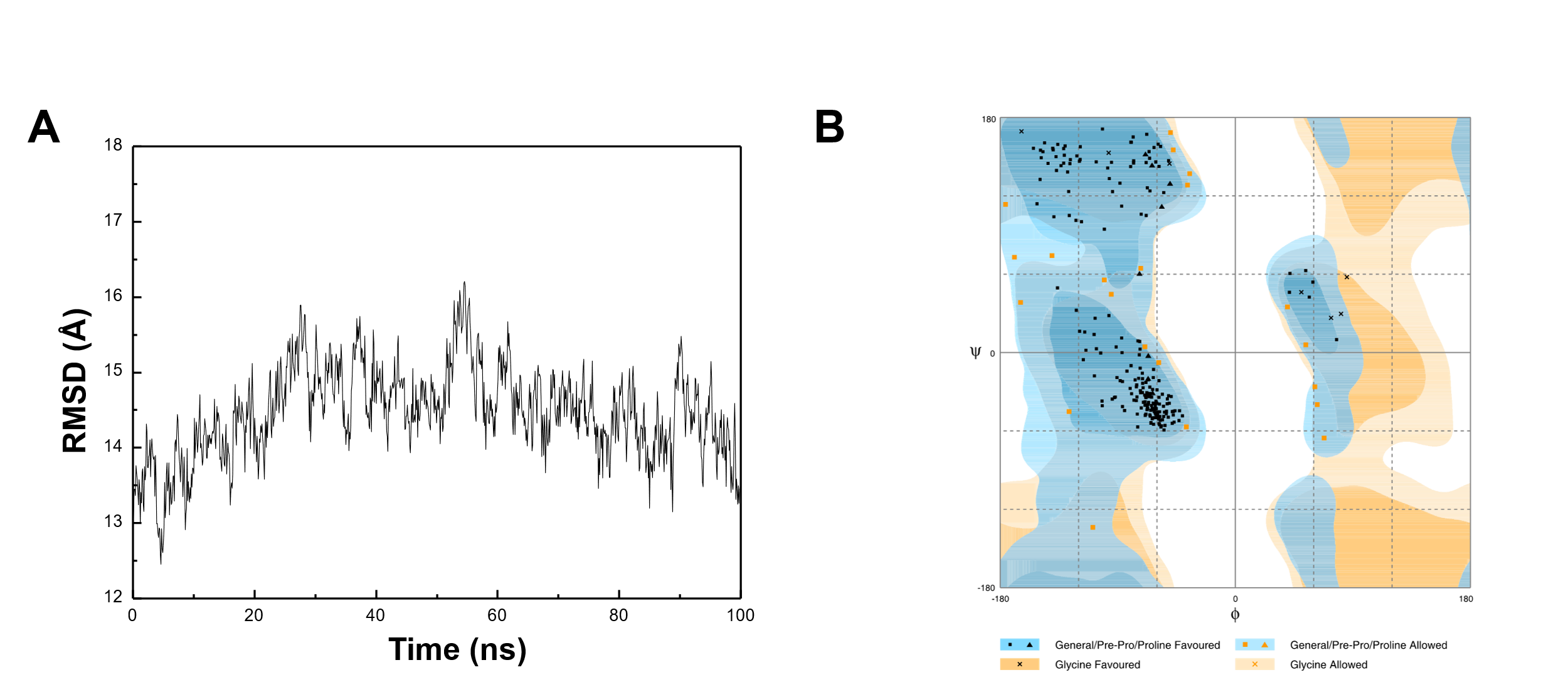


**Supplementary Figure 1.** MD simulations and structural conformations of the *V. fischeri* LuxR. (A) Root-mean-square deviation (RMSD)-time curve for LuxR in 100-ns molecular dynamics (MD) simulation, indicating the stability of the structure. The reference structure used for the RMSD calculation is the TraR crystal structure. (B) Ramachandran plot analysis of LuxR structure. The *x*-axis and *y*-axis represent the values of the φ and ψ backbone dihedral angles, respectively. Dark-blue and light-blue regions respectively represent the favored and allowed regions for general amino acids/pre-Pro/proline. The dark-yellow and light-yellow regions respectively represent the favored and allowed regions for glycine.


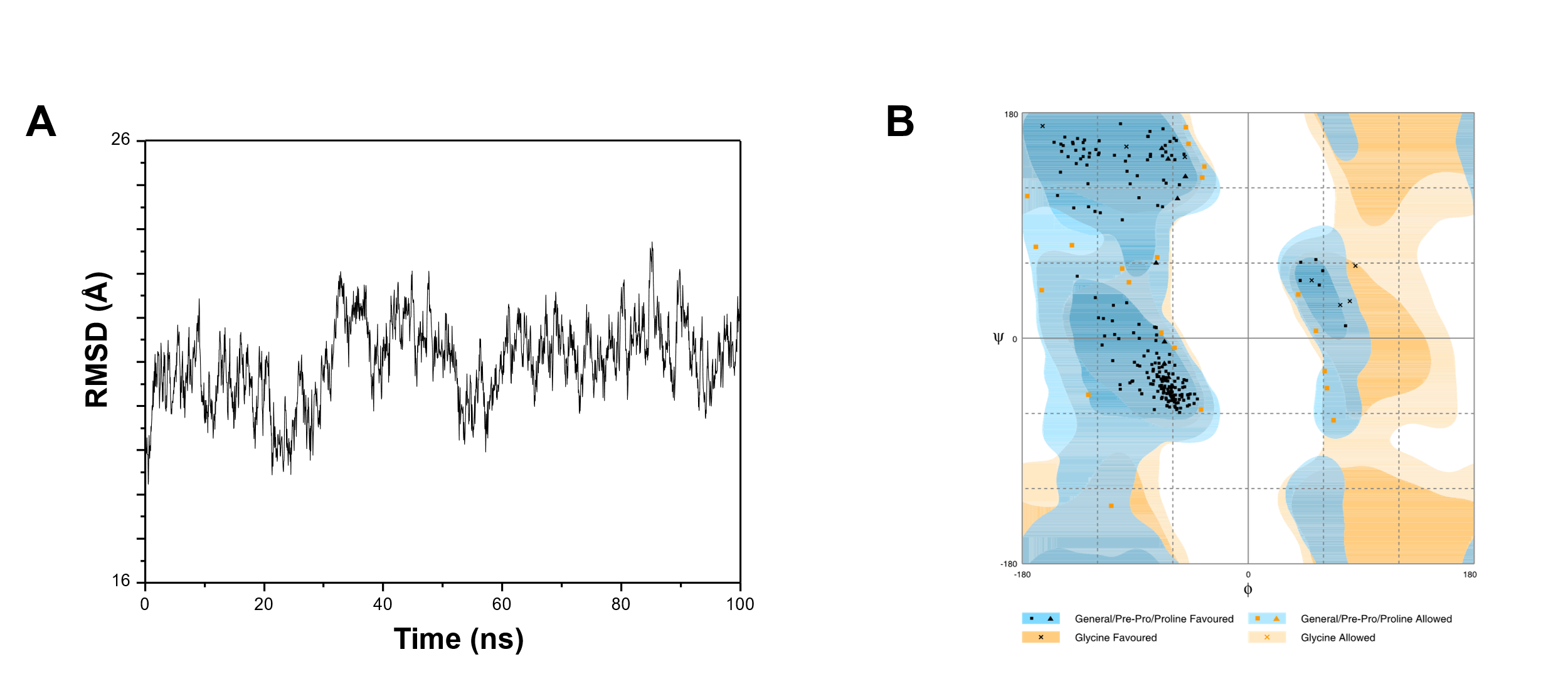


**Supplementary Figure 2.** MD simulations and structural conformations of the *V. fischeri* LuxR–3OC6-HSL complex. (A) RMSD-time curve for LuxR–3OC6-HSL in 100-ns MD simulation, indicating the stability of the LuxR–3OC6-HSL structure. The reference structure used for the RMSD calculation is the TraR crystal structure. (B) Ramachandran plot analysis of the LuxR–3OC6-HSL complex (coloring as in Supplementary Figure 1).


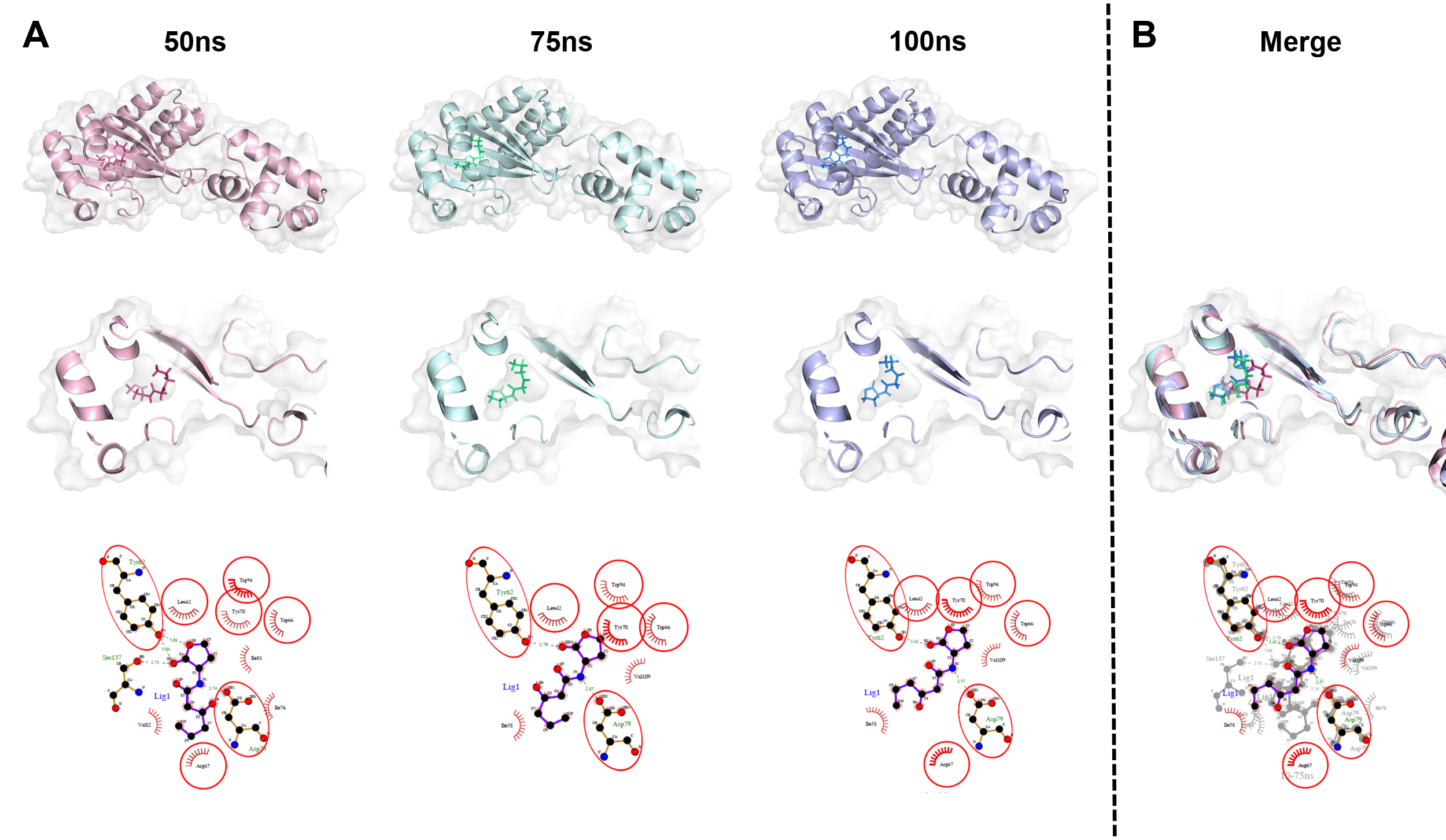


**Supplementary Figure 3.** (A) The overall structure of the LuxR–3OC6-HSL complex at 50, 75, and 100 ns during the MD process. The bottom of the figure shows the key residues that form hydrophilic interactions and hydrogen bonds with 3OC6-HSL. (B) Superposition of the three structural states and corresponding interaction profiles shown in panel A.


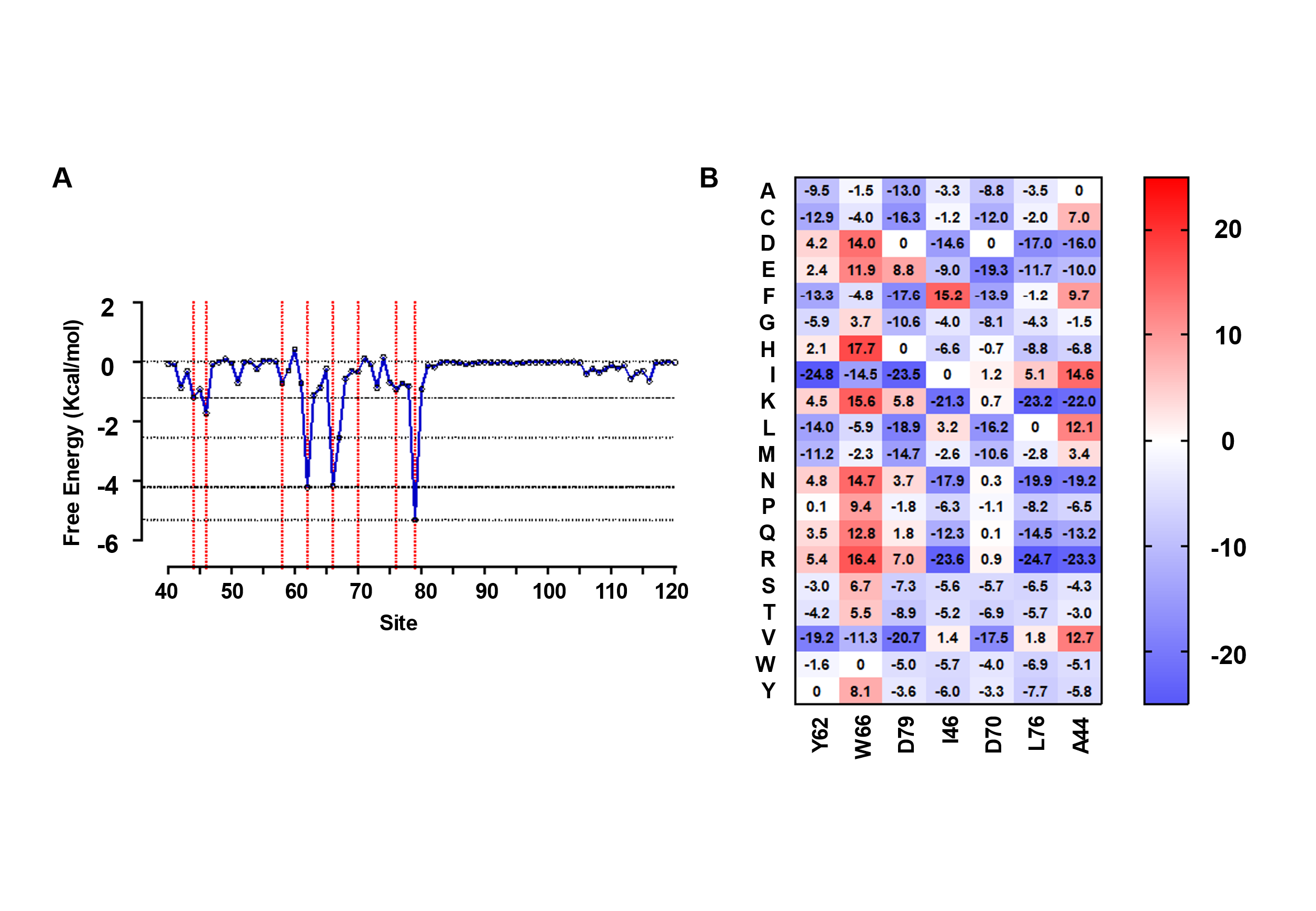


**Supplementary Figure 4.** (A) The free energy for binding of 3OC6-HSL to key sites of *V. fischeri* LuxR. (B) Heatmap of 3OC6-HSL docking energy calculation results with single-residue mutations of LuxR. The *x*-axis indicates the mutation position. The *y*-axis indicates the naturally occurring amino acids that were substituted for the native amino acid in the calculation. Different colors represent different calculated values of binding energy.


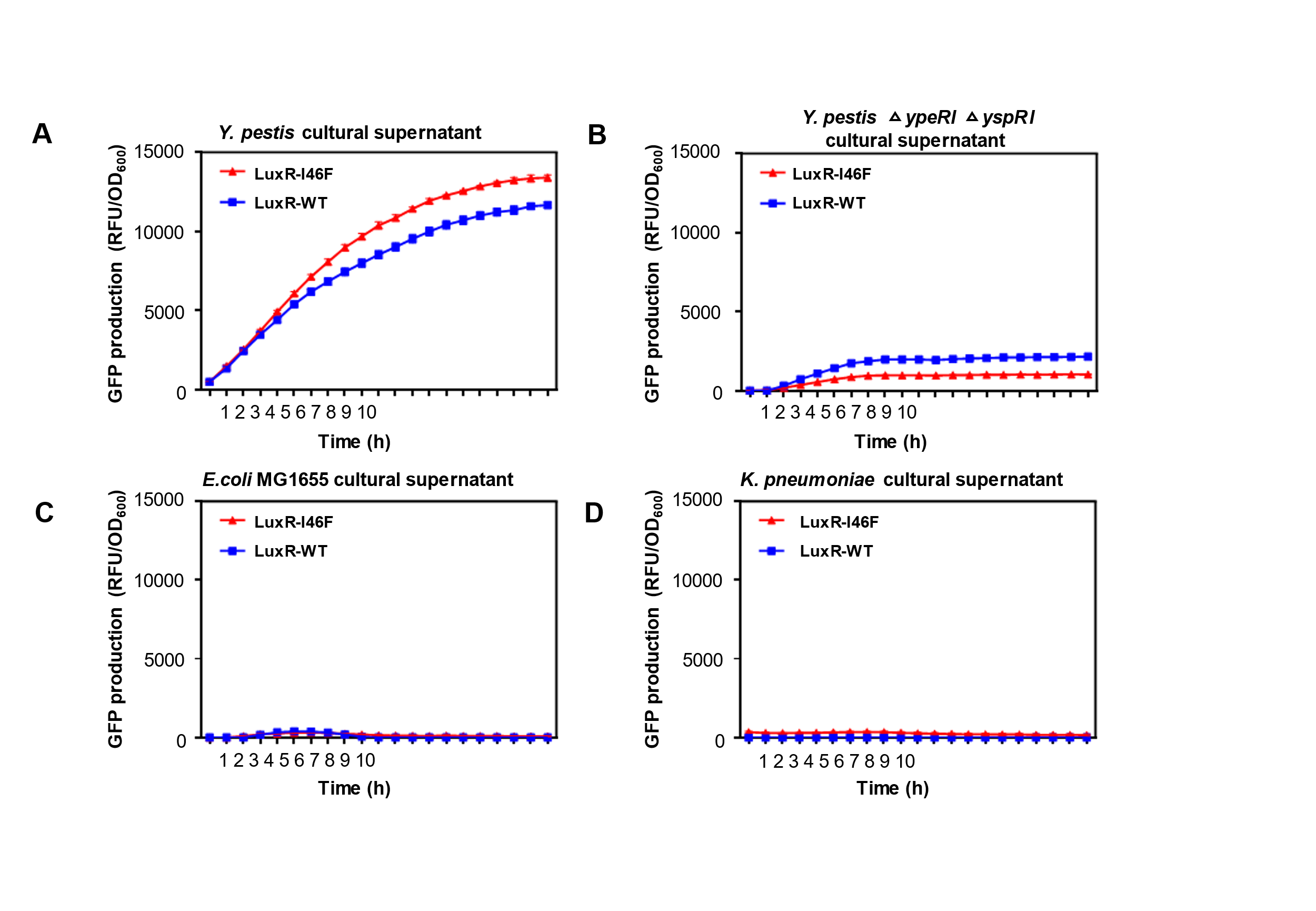


**Supplementary Figure 5.** Curves of relative fluorescence intensity change with temperature. The biosensors based on WT-LuxR and LuxR-I46F were incubated in culture supernatant of *Yersinia pestis* (A)*, Y. pestis* Δ*ypeRI* Δ*yspRI* (B)*, Klebsiella pneumoniae* (C), or *Escherichia coli* strain MG1655 (D). The results are the means of triplicate experiments.


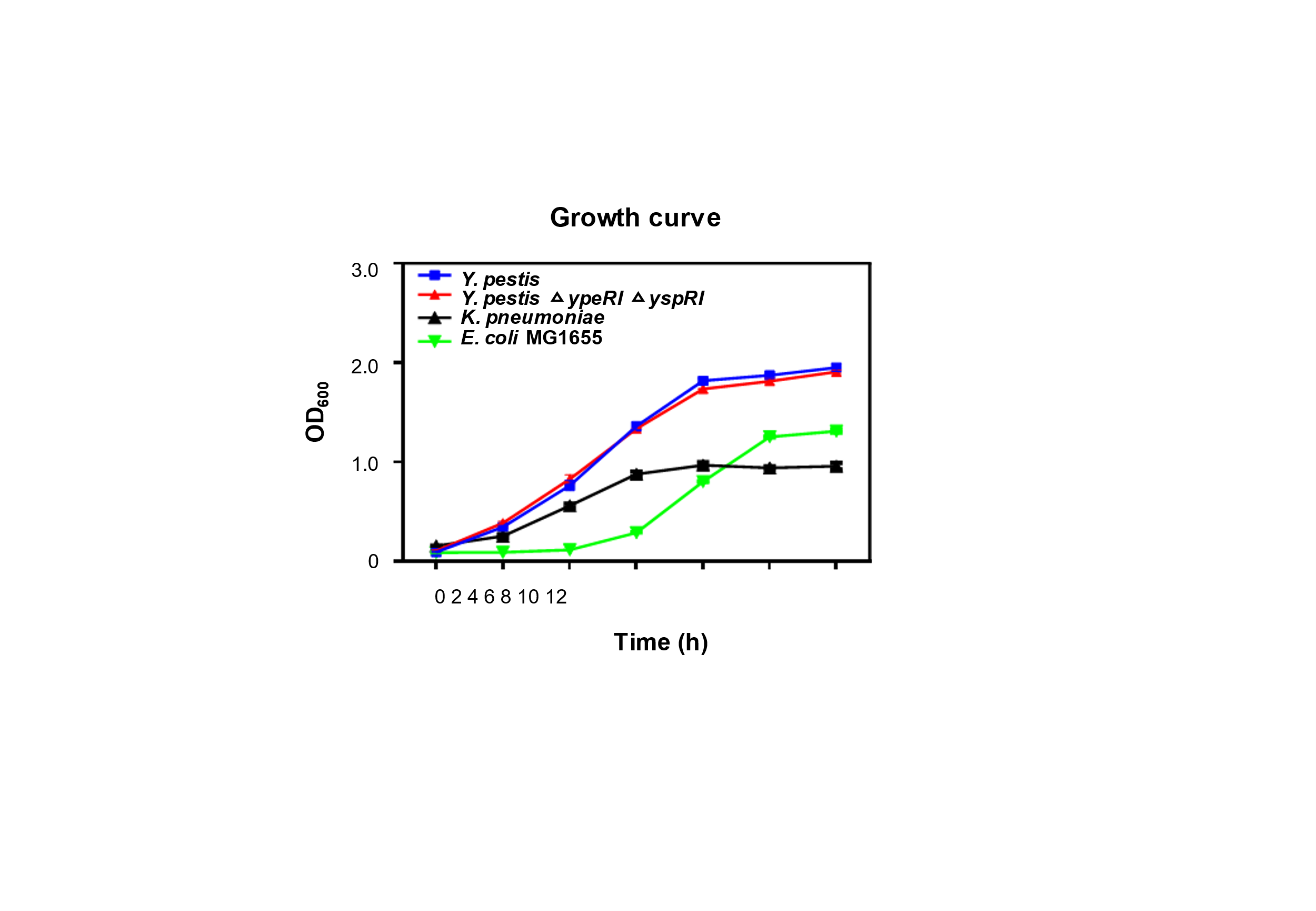


**Supplementary Figure 6.** Growth curves for *Y. pestis*, *Y. pestis* Δ*ypeRI* Δ*yspRI, K. pneumoniae*, and *E. coli* MG1655 grown for 12 h.

**Supplementary Methods**

*Chemicals and bacterial growth conditions*

3OC6-HSL was purchased from Sigma-Aldrich (St. Louis, MO, USA). Stock solutions were prepared by dissolving appropriate amounts of the compound in nuclease-free water and were stored at −40°C.

*Escherichia* *coli* strains were cultured at 37°C in Luria Bertani (LB) medium or on LB-agar plates with 50 or 100 μg/mL ampicillin. For assay using the gene circuit, fresh LB medium (5 mL) supplemented with ampicillin was inoculated with a single *E. coli* colony carrying the plasmid encoding the circuit (see section 2.4 of the main manuscript). Cells were grown overnight at 37°C with shaking at 220 rpm.

*Site-directed mutagenesis*

The coding sequence of the *N*-terminal ligand-binding domain of *Vibrio fischeri* LuxR, spanning residues 1−179, was PCR-amplified from a genomic DNA library. It was then fused directly to the maltose-binding protein (MBP)-encoding sequence in vector pMAT9S. The last three residues of MBP (Asn–Ser–Ser) were changed to Arg–Ile–Thr to ensure the integrity and rigidity of the final helix. This constrains the flexibility of LuxR_1−179_ and solves the precipitation problem commonly observed for LuxR family proteins in the absence of HSL ligands.

LuxR single-site mutants (LuxR-I46F and LuxR-I46R) were constructed by site-directed mutagenesis using a Mut Express II Fast Mutagenesis Kit V2 (Vazyme, Nanjing, China). Briefly, pMAT9S expressing the wild-type LuxR (LuxR-WT)–MBP fusion served as the template for amplification, and Phanta Max Super-Fidelity DNA Polymerase was used (Vazyme, cat. no. P505-d1). Point mutations were introduced using complimentary primers. PCR products were then subjected to digestion by *Dpn*I to remove the template, cyclized using ClonExpress Exnase II (Vazyme, cat. no. C112-01) via homologous recombination, and transformed into *E. coli* TOP10 competent cells. Plasmids extracted from colonies on LB-agar plates were verified by DNA sequencing.
